# Challenges in the Diagnosis and Management of the Miller Fisher Variant of Guillain-Barré Syndrome: A Case Report

**DOI:** 10.7759/cureus.81630

**Published:** 2025-04-02

**Authors:** Evan Ehsan, Angie Posada, Stephen Fletcher

**Affiliations:** 1 Emergency Medicine, William Carey University College of Osteopathic Medicine, Hattiesburg, USA; 2 Neurology, Merit Health Wesley, Hattiesburg, USA; 3 Internal Medicine, Merit Healthy Wesley, Hattiesburg, USA

**Keywords:** acute ataxia, acute demyelinating inflammatory polyneuropathy, deep tendon areflexia, guillain-barré syndrome (gbs), miller fisher syndrome

## Abstract

Miller Fisher syndrome (MFS) is a rare variant of Guillain-Barré syndrome (GBS), characterized by the triad of ophthalmoplegia, ataxia, and areflexia, often following a bacterial or viral illness. This case report presents a 58-year-old Hispanic female patient with a preceding streptococcal pharyngitis who developed symptoms of bilateral facial nerve palsy and lower limb ataxia. Cerebrospinal fluid (CSF) analysis revealed albuminocytologic dissociation and, combined with her presentation of ophthalmoplegia, ataxia, and areflexia, supported the diagnosis of MFS. Magnetic resonance imaging (MRI) and laboratory tests were performed, revealing nonspecific findings and a lack of evidence for alternative diagnoses. Despite the absence of electrodiagnostic studies or GQ1b antibody testing, intravenous immunoglobulin was initiated based on clinical suspicion. The patient's symptoms slowly improved, with the prognosis suggesting a need for long-term rehabilitation. This case highlights an unusual presentation of MFS, complicated by Raynaud's phenomenon and a rare infectious trigger of* Streptococcus pyogenes*, emphasizing the need for further research on atypical MFS presentations and the role of adjunct therapies in management.

## Introduction

Guillain-Barré syndrome (GBS) encompasses several types of acute immune-mediated polyneuropathies, the most common being acute inflammatory demyelinating neuropathy [1]. Clinically, GBS typically presents as rapidly evolving ascending weakness, mild sensory loss, and hypo- or areflexia that reaches its physiologic nadir in about four weeks [2]. GBS is considered an autoimmune disease, typically triggered by a preceding bacterial or viral infection. The most common infectious triggers are *Campylobacter jejuni* and *Haemophilus influenzae*, while less common causes include cytomegalovirus, Epstein-Barr virus, and human immunodeficiency virus (HIV) [2,3]. Epidemiologically, GBS has a median annual occurrence of 1.3 cases per population of 100,000, with men being affected more than women [3]. Observed in only 1-5% of all cases of GBS in Western countries [4], Miller Fisher syndrome (MFS), a rare variant of GBS, typically presents with two of the following: ataxia, areflexia, and ophthalmoplegia in the setting of preceding bacterial or viral illness. MFS is commonly associated with the involvement of the lower cranial nerves and facial nerves and does not usually involve motor weakness of the limbs [2]. 

If there is clinical suspicion of MFS and/or GBS, a lumbar puncture with appropriate cerebrospinal fluid (CSF) studies is warranted to narrow the differential diagnosis. In 90% of cases of MFS/GBS, there is an albuminocytologic dissociation, which is defined as a raised CSF protein level with a normal cell count. However, the absence of this dissociation does not exclude MFS/GBS as a differential diagnosis [2]. Electrodiagnostic studies may show reduced or absent sensory responses without slowing of sensory conduction studies [5]. Magnetic resonance imaging (MRI) of the brain may show enhancement of the oculomotor, abducens, and facial nerves [6]. MRI of the spine may reveal thickening and enhancement of the intrathecal spinal nerve roots and cauda equina and/or lesions confined to the spinal posterior columns responsible for the ataxia [7]. Lastly, serum antibodies against GQ1b (a ganglioside component of nerve) are present in 85-90% of patients with MFS [8].

This case report examines the challenges in diagnosing and managing the Miller Fisher variant of GBS in a 58-year-old Hispanic female patient who presented with lower leg ataxia and facial nerve palsy. Throughout her care, we discuss the approaches to symptomatic relief, relevant clinical procedures, and lab findings supporting the diagnosis of MFS. Due to limited evidence on the diverse presentations of MFS, we discuss two atypical findings in this patient: Raynaud's phenomenon and a possible autoimmune etiology of *Streptococcus pyogenes*.

## Case presentation

A 58-year-old Hispanic female patient presented to the emergency department (ED) with a chief complaint of weakness. One week prior to admission, the patient was diagnosed with strep throat and sent home with amoxicillin-clavulanate. Five days prior to admission, she noticed weakness in her face and fell abruptly due to sudden-onset bilateral lower extremity weakness; she stated she "lost control" of her legs. She went to the ED at another local hospital and received a computed tomography (CT) of the head without contrast, which showed no acute abnormalities. She was found to have hypomagnesemia and was admitted overnight at that hospital for treatment. Over the course of the next few days, she developed left-sided facial droop, tingling in her hands, and lower extremity weakness, numbness, and tingling. She had difficulty rising from a chair due to hand and leg weakness, as well as had pain in multiple joints and in her lower extremities. She had been having periodic night sweats over the past few months, which she described as similar to going through menopause, although she was postmenopausal. Her last bowel movement was four days prior to admission. She had no shortness of breath, weight loss/gain, or rash and denied recent travel or tick or insect bites. She was an active smoker with a 30-year smoking history. She also admitted to past methamphetamine and cocaine use but had been drug-free for the past 24 years. She stated that nothing like this had ever happened before. 

In our ED, physical examination included the following vital signs: heart rate of 91, respiration rate of 19, blood pressure of 159/88, oxygen saturation at 98% on room air, and body mass index (BMI) of 40.2. Exam findings showed bilateral lower extremity weakness with areflexia and full sensation, rapid horizontal gaze nystagmus when looking left to right, unable to smile, muffled speech, difficulty closing eyes, and mild pharyngeal erythema. No signs of trauma from her fall were noted. No signs of respiratory distress were noted. 

An initial CT of the head was performed, with no acute intracranial hemorrhage or masses seen. Laboratory investigations were ordered, including a complete blood count (CBC), complete metabolic panel (CMP), erythrocyte sedimentation rate (ESR), C-reactive protein (CRP), vitamin B12, and folic acid (Table 1). Pertinent lab findings revealed leukocytosis, elevated mean corpuscular hemoglobin (MCH), elevated mean corpuscular volume (MCV), thrombocytosis, elevated serum protein, elevated ESR, elevated CRP, elevated alkaline phosphatase, and elevated vitamin B12. A lumbar puncture was performed and revealed albuminocytologic dissociation (Table 2).

**Table 1:** Laboratory findings initially indicating leukocytosis, elevated MCH, elevated MCHC, elevated protein, elevated ESR, elevated CRP, elevated alkaline phosphatase, and elevated vitamin B12. Over time, WBC and platelets trended downward toward normal, suggesting resolution of an inflammatory process. Electrolyte imbalances, including hypokalemia, improved, while total protein and globulin remained elevated, likely due to IVIg infusion. Alkaline phosphatase gradually decreased but stayed mildly elevated. WBC: white blood cells; Hb: hemoglobin; Hct: hematocrit; MCH: mean corpuscular hemoglobin; MCHC: mean corpuscular hemoglobin concentration; MCV: mean corpuscular volume; ALT: alanine transaminase; AST: aspartate transaminase; TSH: thyroid-stimulating hormone; T4: thyroxine; IVIg: intravenous immunoglobulin

Test	Day 4	Day 3	Day 2	Day 1	Reference range
WBC	5.8×10³/μL	9.1×10³/μL	8.8×10³/μL	12.5×10³/μL	4.0-11.0×10³/μL
Hb	12.3 g/dL	13.0 g/dL	12.9 g/dL	14.6 g/dL	12.1-15.1 g/dL
Hct	37.50%	38.90%	39.50%	43.90%	42-52%
MCH	33.2 pg/cell	33.9 pg/cell	33.1 pg/cell	33.5 pg/cell	25-35 pg/cell
MCHC	32.8% Hb/cell	33.4% Hb/cell	32.6% Hb/cell	33.2% Hb/cell	31-36% Hb/cell
MCV	101.2 fL	101.3 fL	101.4 fL	101 fL	80-100 fL
Platelet count	367×10⁹/L	393×10⁹/L	383×10⁹/L	500×10⁹/L	150-450×10⁹/L
Sodium	136 mmol/L	137 mmol/L	136 mmol/L	138 mmol/L	135-147 mmol/L
Potassium	3.8 mmol/L	2.9 mmol/L	3.4 mmol/L	3.7 mmol/L	3.5-5.0 mmol/L
Chloride	108 mmol/L	106 mmol/L	106 mmol/L	106 mmol/L	96-106 mmol/L
Calcium	8.5 mg/dL	8.9 mg/dL	8.9 mg/dL	9.7 mg/dL	8.4-10.2 mg/dL
Carbon dioxide	24 mmol/L	26 mmol/L	23 mmol/L	23 mmol/L	23-29 mmol/L
Blood urea nitrogen	9 mmol/L	9 mmol/L	10 mmol/L	17 mmol/L	2.1-8.5 mmol/L
Creatinine	0.55 mg/dL	0.70 mg/dL	0.65 mg/dL	0.89 mg/dL	0.6-1.2 mg/dL
Glucose	103 mg/dL	143 mg/dL	92 mg/dL	93 mg/dL	<140 mg/dL
Alkaline phosphatase	105 U/L	112 U/L	121 U/L	134 U/L	25-125 U/L
ALT	15 U/L	17 U/L	17 U/L	21 U/L	10-40 U/L
AST	15 U/L	16 U/L	18 U/L	22 U/L	12-38 U/L
Bilirubin, total	0.3 mg/dL	0.3 mg/dL	0.8 mg/dL	0.6 mg/dL	0.1-1.0 mg/dL
Creatine kinase	-	-	-	108 U/L	10-70 U/L
Protein, total	8.9 g/dL	8.3 g/dL	8.1 g/dL	8.3 g/dL	6.0-7.8 g/dL
Albumin	3.5 g/dL	3.6 g/dL	3.7 g/dL	4.5 g/dL	3.5-5.5 g/dL
Globulin	5 mmol/L	5 mmol/L	4 mmol/L	4 mmol/L	2.3-3.5 mmol/L
Vitamin B12	-	-	-	1207 pg/mL	118-701 pg/mL
Folate	-	-	-	13.5 ng/mL	2.7-17.0 ng/mL
Magnesium	-	-	2.1 mg/dL	2.3 mg/dL	1.5-2.0 mg/dL
TSH	-	-	-	1.4 mIU/mL	0.4-4.0 mIU/mL
T4, free	-	-	-	1.4 ng/dL	0.9-1.7 ng/dL

**Table 2:** Lumbar puncture revealing albuminocytologic dissociation with normal WBCs and elevated total protein. RBC: red blood cells; WBC: white blood cells; CSF: cerebrospinal fluid

Test	Observed value	Reference range
RBC	3/uL	<1/uL
WBC	3/uL	0-5/uL
CSF clarity	Clear	Clear
CSF color	Colorless	Colorless
Glucose	62 mg/dL	50-75 mg/dL
Protein, total	183.1 mg/dL	14-45 mg/dL

With the albuminocytologic dissociation on CSF studies and physical exam findings, GBS was the top differential diagnosis. With her smoking history, Lambert-Eaton myasthenic syndrome was in the differential as well as stenotic changes. Autoimmune etiologies such as multiple sclerosis, polymyalgia rheumatica, dermatomyositis, and polymyositis were considered with the elevated inflammatory markers; however, creatine kinase (CK) was not significantly elevated. Infectious etiologies such as hepatitis and HIV were also considered.

Given inconsistent physical exam findings and without a definitive diagnosis, the patient was admitted to the Medicine floor for further evaluation and management. Given the albuminocytologic dissociation observed in CSF studies and the patient's clinical presentation, which strongly suggested acute inflammatory demyelinating polyneuropathy (AIDP), intravenous immunoglobulin (IVIg) at a dose of 400 mg/kg for five days was initiated according to the standard treatment regimen to optimize patient outcomes. Additionally, acetylcholine receptor antibodies were ordered to rule out myasthenia gravis. The patient's sensory changes could have been attributed to the neurologic manifestation of thiamine deficiency; thus, she was started on IV thiamine supplementation. Due to her persistent pharyngeal erythema, a course of ceftriaxone was initiated as she may not have completed her course of amoxicillin-clavulanate. 

A teleneurology service was consulted per the hospital's protocol, and the teleneurologist supported GBS as the diagnosis and advised to continue the IVIg. MRI with and without contrast of the brain, cervical spine, and lumbar spine were ordered to look for potential causes including demyelinating disease such as multiple sclerosis and transverse myelitis. MRI of the thoracic spine with and without contrast were omitted due to patient distress in the MRI machine and low suspicion for malignancy. MRI of the brain without contrast showed a few small white matter hyperintensities in the frontal lobes, which were nonspecific and attributed to migraine headaches or mild chronic microvascular disease. MRI of the lumbar spine without contrast showed an incidental atrophic right kidney, confirming the patient's history of stent-related kidney damage. All the remaining MRIs were unremarkable.

On day 2 of admission, the patient seemed to have some improvement in muscle strength and right patellar reflex changing from 0/4 to 1/4. Hepatitis C (HCV) antibody screening and HIV antigen/antibody screening were negative. CSF studies were negative for *Haemophilus*, *Neisseria*, group B strep, cryptococcus, and meningococcus. 

On day 3, there was no improvement with her bilateral facial nerve deficits, ascending asymmetric weakness, or dysesthesias in bilateral lower extremities. Her speech was less muffled, but she still had difficulty pronouncing certain words due to upper lip weakness. Her muscle strength increased through serial exams throughout the day. Laboratory studies showed hypokalemia (3.7 to 2.9) and vitamin D deficiency, for which potassium chloride and ergocalciferol were initiated, respectively. Serum protein levels continue to be elevated, most likely due to the IVIg. A chest CT was ordered to rule out thymoma or small cell lung cancer as the cause of potential myasthenia gravis and Lambert-Eaton as a paraneoplastic syndrome, respectively. No acute abnormalities or masses were found on the chest CT. Her urinalysis was positive for barbiturates. 

On day 4, she had full flexion of her left hip and 4/5 strength of her right hip, being inconsistent with GBS. Her upper lip was more swollen that day, and diphenhydramine was prescribed. Later that night, she had an episode of vaso-occlusion in her distal digits of the hands and feet with pallor, most likely Raynaud's phenomenon. She was started on gabapentin, after which these symptoms resolved. 

On day 5, the teleneurology consultant stated that this was a classic presentation of AIDP and, with bilateral facial nerve palsy, likely represented the Miller Fisher variant of AIDP. At that point, the patient had a full five-day course of IVIg and had mildly improved strength and speech, which was all we could have expected at the time. AIDP levels off by days 7-10, and there is a long road to recovery if the patient is healthy enough to recover. 

The patient would need several months of rehabilitation and follow-up with an outpatient neurologist for electromyography (EMG) and nerve conduction studies (NCS) to assess the improvement. A nighttime eyepatch was recommended to prevent corneal abrasions due to bilateral facial nerve palsy, alternating eyes each night and wearing the eyepatch when outside or windy. Lubricating eye drops were recommended on a daily basis to prevent drying out of the cornea. The patient was discharged to a rehabilitation facility on day 6. 

Following her discharge, send-out labs exploring the causes of the patient's polyneuropathy resulted as follows: antinuclear antibody screen was negative; rapid plasma reagin (RPR) was non-reactive, ruling out syphilis; and acetylcholine receptor-binding, receptor-blocking, and receptor-modulating antibodies were negative, ruling out myasthenia gravis. Herpes simplex viruses (HSV) 1 and 2 were negative in the CSF, ruling out herpes encephalitis. Further exploring the etiology of the polyneuropathy, there was no M spike, ruling out multiple myeloma. Myelin basic protein in the CSF was negative, which can be elevated in acute disseminated encephalitis. We also ruled out mononeuropathy multiplex secondary to vasculitis, and myeloperoxidase and proteinase-2 antibody tests were negative. Her thiamine levels were within normal limits, and her IgG, IgA, and IgM levels were elevated in the CSF, in a chronic inflammatory pattern.

## Discussion

MFS is a rare variant of GBS, characterized by the triad of clinical symptoms: ophthalmoplegia, ataxia, and areflexia. Approximately one-quarter of patients with MFS also develop limb weakness, as did our patient [9,10]. This condition typically follows a bacterial or viral infection, and it is caused by the body's immune system mistakenly attacking peripheral nerves due to molecular mimicry. Most patients have antibodies against GQ1b, a ganglioside found in nerves, which is believed to be the key pathogenic mechanism [1]. Unlike classical GBS, MFS often spares muscle strength. 

Diagnostic evaluation includes the clinical history of the classic triad of symptoms and preceding illness, CSF analysis, MRI, electrodiagnostic studies such as EMG and NCS, and the presence of anti-GQ1b antibodies. The clinical history of ophthalmoplegia (eye muscle paralysis), ataxia (impaired coordination), and areflexia (absent deep tendon reflexes) is critical to the diagnosis of MFS. Other differentials include GBS, which would present with significant ascending limb weakness, and Bickerstaff brainstem encephalitis, which would usually exhibit altered consciousness and/or hyperreflexia. In this case, the patient presented with the classic triad of symptoms following a streptococcal pharyngeal infection, indicating high suspicion for MFS. Her asymmetric weakness made this an atypical presentation of MFS, adding to the diagnostic challenge, as it is rarely discussed in the literature. 

CSF analysis typically shows albuminocytologic dissociation, which is described as a combination of elevated protein levels yet normal white blood cell count [11]. However, albuminocytologic dissociation is not diagnostic for MFS nor GBS, and this dissociation identifies a broad range of neurological diagnoses, including non-inflammatory polyneuropathy, hydrocephalus, and encephalitis. In this case, albuminocytologic dissociation paired with the clinical history further raises suspicion for MFS. Serum protein levels continued to remain elevated despite IVIg treatment due to the immunoglobulin being a protein, falsely elevating the serum protein level.

MRI is usually normal in MFS but may be performed to rule out other causes of ophthalmoplegia or ataxia such as stroke, multiple sclerosis, or transverse myelitis. A spinal MRI may reveal the thickening and enhancement of the intrathecal spinal nerve roots and cauda equina, with the anterior spinal nerve roots selectively involved. In exceptional cases of MFS, abnormalities of the posterior columns have been described. On brain MRI, enhancement of certain cranial nerves, such as the oculomotor, abducens, and facial nerves, may be seen [12]. In this case, MRI with and without contrast of the brain, cervical spine, and lumbar spine did not show any abnormalities of the cranial nerves or spinal cord. 

Electrodiagnostic studies may show reduced or absent sensory responses without slowing of sensory conduction studies [2]. EMG can be particularly helpful in excluding alternative differential diagnoses which would show no abnormalities, such as ocular myasthenia gravis and Lambert-Eaton myasthenic syndrome. This equipment is not available at our inpatient facility. Thus, these tests were deferred for the outpatient follow-up.

Autoantibody testing can be a crucial confirmatory test of MFS, with serum IgG antibodies to GQ1b having a sensitivity of 85-90% [8]. Particularly, biochemical analysis showed that certain cranial nerves contained a larger amount of GQ1b than did the ventral and dorsal roots of the spinal cord, which could explain the ophthalmoplegic component of MFS [8]. GQ1b antibodies along with the corresponding clinical history must be present to confirm diagnosis, as GQ1b antibodies can be present in GBS and other variants such as Bickerstaff brainstem encephalitis [13]. In this case, the clinical presentation, presence of albuminocytologic dissociation, and absence of imaging abnormalities strongly supported the diagnosis of MFS. GQ1b antibody testing was foregone for financial considerations. 

MFS is largely treated with adequate supportive care, pain control, respiratory support as needed, and immunotherapy. In the past, oral or IV steroids were used in the treatment of GBS or MFS but are no longer recommended due to their ineffectiveness [2]. IVIg and plasmapheresis are both effective treatments for GBS and severe cases of MFS, with no difference in outcomes when treatments are compared. Most patients with MFS usually do not require immunotherapy due to their strong prognosis and spontaneous recovery. IVIg is typically warranted for more severe cases of MFS with signs of respiratory distress. Before IVIg therapy is initiated, clinicians should check serum IgA levels because IgA-deficient patients are at higher risk of anaphylaxis [2]. The usual IVIg dose is 2 g/kg divided over 2-5 days with a second treatment course that may be necessary. Alternatively, 400 mg/kg per dose IV daily for five days has been used. Plasmapheresis (2-3 L of plasma/body weight) over two weeks is the standard course for those patients who are unable to ambulate without assistance. Mildly affected patients with a low disability score still benefit from 2 plasma exchanges of 1.5 plasma volumes [2]. Contraindications for plasma exchange include recent use of IVIg (immunoglobulins could be filtered out, potentially negating their benefits), hemodynamic instability, pregnancy, sepsis, and hypocalcemia [2]. In this case, IVIg therapy was initiated on day 1 due to the clinical suspicion of GBS and MFS. Treatment showed mild resolution of her symptoms, with muscle strength slightly increasing through serial exams and patellar reflex going from 0/4 to 1/4, as expected due to the limited use of IVIg for milder cases of MFS. Supportive care was provided as well as pain control, including gabapentin for her vaso-occlusive episode and acetaminophen for generalized pain. The patient showed no signs of respiratory distress; thus, no respiratory treatments were initiated. 

Typically, several months of intense physiotherapy programs are critical to recover neurological function, as neurons grow 1 mm a day [14]. An appropriate plan of care can help patients minimize pain, increase strength and endurance, and prevent secondary complications including overuse damage to muscles and joints while improving balance and mobility and restoring functional activity [2]. Patients with MFS can typically begin physical and occupational therapy (PT/OT) inpatient, continue therapy at a nursing/rehab facility such as skilled nursing facilities, and eventually translate to home-based therapies [2]. In this case, PT/OT was initiated on day 1 of her inpatient stay, which contributed to the mild resolution of her ataxia. PT and OT were performed throughout her stay, and these continued at the skilled nursing facility to which she was discharged. After two weeks at the skilled nursing facility, she was able to move her upper lip and regain some of her strength in her right leg. We expect to see continued recovery through consistent rehabilitation. 

The prognosis for MFS is usually good with a case fatality of less than 5%. The mean recovery times range from eight to 12 weeks, with residual symptoms sometimes being present in some patients. Although recurrence may occur, it is uncommon [2].

A bacterial or viral illness typically precedes MFS and GBS, theorized to be due to the body confusing the nerves with infectious proteins due to molecular mimicry, which leads to nerve damage. Temporally, MFS occurs 12 hours to 28 days after the onset of the initial triggering infection [2]. The most common bacterial trigger has been *Campylobacter jejuni*, and the most common viral triggers have been HIV, Epstein-Barr virus, and Zika virus [2]. In a study, sore throat was statistically more frequent in MFS (18/24 cases, 75%) than in GBS (29/58, 50%). However, in series, there was no association of MFS with group A streptococcal infection. Some patients may develop MFS after streptococcal pharyngitis, but this causative bacterium has not shown to be a major antecedent agent in MFS [15]. In this case, MFS did develop following the patient's recent history of streptococcal pharyngitis, a less common cause. Prevention of MFS is solely limited to the prevention of the antecedent infectious agent, which includes improved hygiene practices, cooking poultry products thoroughly, and disinfecting the water supply [2]. As there are currently no screening guidelines, risk factors include the use of certain drugs (heroin, suramin, streptokinase, and isotretinoin), use of TNF-alpha antagonist therapy, other concurrent autoimmune diseases, surgery, epidural anesthesia, bone marrow transplant, and immunizations [2].

Despite the comprehensive evaluation and treatment plan, there are some limitations in this case. First, electrodiagnostic studies were not performed as the equipment is not available at this inpatient facility. EMG and NCS could have helped confirm or exclude alternative diagnoses such as myasthenia gravis, Lambert-Eaton myasthenic syndrome, or other neuromuscular disorders. The absence of these studies limits the strength of the diagnostic conclusions in this case. Additionally, GQ1b antibody testing was not ordered, which is a crucial diagnostic tool for MFS. Although the clinical presentation, CSF findings, and imaging results supported the diagnosis, GQ1b antibodies are highly sensitive and could have provided a more definitive confirmation. With the sensitivity reported to be 85-90%, their absence or presence would have strengthened the diagnosis. By not including the antibody testing, this case misses an opportunity to firmly establish the diagnosis and fully explore the relationship between streptococcal pharyngitis and MFS, especially given the rarity of such an association. The patient's positive drug screen for barbiturates, despite her denying illicit drug use, may be explained by her use of ibuprofen, which is known to cause false positives on urine drug tests [16]. Lastly, while the patient's response to IVIg was noted to show mild improvement in muscle strength and reflexes, the lack of detailed follow-up limits the assessment of long-term outcomes. The early improvements noted may not fully reflect the patient's recovery trajectory over time.

## Conclusions

This case illustrates an interesting variant presentation of the Miller Fisher variant of GBS, with the classic symptoms marked by bilateral facial nerve palsy, ataxia, and areflexia following a recent infection, although the patient had asymmetric weakness which varied from day to day. The patient's clinical progression, supported by the albuminocytologic dissociation in CSF analysis, led to the initiation of IVIg therapy. Although the patient showed slow but steady progress, full recovery typically takes several months of rehabilitation. Future outpatient evaluations, including NCS and EMG, will be pertinent in monitoring her long-term recovery and guiding further management. The presence of Raynaud's phenomenon and preceding infection with *Streptococcus pyogenes* make this case more unique and further warrant research regarding clinical presentations of MFS due to its variability. Additionally, further studies are needed to explore the role of adjunct therapies in mitigating symptoms, especially in patients with atypical presentations or those who fail to show immediate improvement. Investigation into the correlation between infectious triggers and autoimmune neuropathies like MFS may also provide insight into prevention and earlier diagnosis.

## References

[1] Dimachkie MM, Barohn RJ (2013). Guillain-Barré syndrome and variants. Neurol Clin.

[2] Rocha Cabrero F, Morrison EH (2023). Miller Fisher syndrome. StatPearls [Internet].

[3] Kuwabara S (2004). Guillain-Barré syndrome: epidemiology, pathophysiology and management. Drugs.

[4] Urlapu KS, Saad M, Bhandari P, Micho J, Hassan MT (2020). Miller Fisher variant of Guillain-Barré syndrome: a great masquerader. Cureus.

[5] Fross RD, Daube JR (1987). Neuropathy in the Miller Fisher syndrome: clinical and electrophysiologic findings. Neurology.

[6] Hattori M, Takada K, Yamada K, Kamimoto K, Mitake S (1999). A case of Miller Fisher syndrome with gadolinium-enhancing lesions in the cranial nerves and the cauda equina on magnetic resonance imaging [Article in Japanese]. Rinsho Shinkeigaku.

[7] Inoue N, Ichimura H, Goto S, Hashimoto Y, Ushio Y (2004). MR imaging findings of spinal posterior column involvement in a case of Miller Fisher syndrome. AJNR Am J Neuroradiol.

[8] Chiba A, Kusunoki S, Obata H, Machinami R, Kanazawa I (1993). Serum anti-GQ1b IgG antibody is associated with ophthalmoplegia in Miller Fisher syndrome and Guillain-Barré syndrome: clinical and immunohistochemical studies. Neurology.

[9] FI M (1956). An unusual variant of acute idiopathic polyneuritis (syndrome of ophthalmoplegia, ataxia and areflexia). N Engl J Med.

[10] Lo YL (2007). Clinical and immunological spectrum of the Miller Fisher syndrome. Muscle Nerve.

[11] Brooks JA, McCudden C, Breiner A, Bourque PR (2019). Causes of albuminocytological dissociation and the impact of age-adjusted cerebrospinal fluid protein reference intervals: a retrospective chart review of 2627 samples collected at tertiary care centre. BMJ Open.

[12] Chandrashekhar S, Dimachkie MM (2024). Guillain-Barré syndrome in adults: pathogenesis, clinical features, and diagnosis. UpToDate.

[13] Lee SU, Kim HJ, Choi JY, Choi KD, Kim JS (2024). Expanding clinical spectrum of anti-GQ1b antibody syndrome: a review. JAMA Neurol.

[14] Baradaran A, El-Hawary H, Efanov JI, Xu L (2021). Peripheral nerve healing: so near and yet so far. Semin Plast Surg.

[15] Yuki N, Hirata K (1998). Fisher's syndrome and group A streptococcal infection. J Neurol Sci.

[16] Algren DA, Christian MR (2015). Buyer beware: pitfalls in toxicology laboratory testing. Mo Med.

